# Establishment of a mortality risk nomogram for predicting in-hospital mortality of sepsis: cohort study from a Chinese single center

**DOI:** 10.3389/fmed.2024.1360197

**Published:** 2024-05-03

**Authors:** Hongsheng Wu, Shichao Jia, Biling Liao, Tengfei Ji, Jianbin Huang, Yumei Luo, Tiansheng Cao, Keqiang Ma

**Affiliations:** ^1^Hepatobiliary Pancreatic Surgery Department, Huadu District People’s Hospital of Guangzhou, Guangzhou, China; ^2^Information Network Center, Huadu District People’s Hospital of Guangzhou, Guangzhou, China

**Keywords:** sepsis, mortality risk, nomogram, cohort study, predictive model

## Abstract

**Objective:**

To establish a mortality risk nomogram for predicting in-hospital mortality of sepsis patients in the Chinese population.

**Methods:**

Data were obtained from the medical records of sepsis patients enrolled at the Affiliated Huadu Hospital, Southern Medical University, between 2019 and 2021. A total of 696 sepsis patients were initially included in our research, and 582 cases were finally enrolled after screening and divided into the survival group (*n* = 400) and the non-survival group (*n* = 182) according to the incidence of mortality during hospitalization. Twenty-eight potential sepsis-related risk factors for mortality were identified. Least absolute shrinkage and selection operator (LASSO) regression was used to optimize variable selection by running cyclic coordinate descent with *k*-fold (tenfold in this case) cross-validation. We used binary logistic regression to build a model for predicting mortality from the variables based on LASSO regression selection. Binary logistic regression was used to establish a nomogram based on independent mortality risk factors. To validate the prediction accuracy of the nomogram, receiver operating characteristic curve (ROC) analysis, decision curve analysis (DCA) and restricted cubic spline (RCS) analysis were employed. Eventually, the *Hosmer-Lemeshow* test and calibration curve were used for nomogram calibration.

**Results:**

LASSO regression identified a total of ten factors, namely, chronic heart disease (CHD), lymphocyte count (LYMP), neutrophil-lymphocyte ratio (NLR), red blood cell distribution width (RDW), C reactive protein (CRP), Procalcitonin (PCT), lactic acid, prothrombin time (PT), alanine aminotransferase (ALT), total bilirubin (Tbil), interleukin-6 (IL6), that were incorporated into the multivariable analysis. Finally, a nomogram including CHD, LYMP, NLR, RDW, lactic acid, PT, CRP, PCT, Tbil, ALT, and IL6 was established by multivariable logistic regression. The ROC curves of the nomogram in the training and validation sets were 0.9836 and 0.9502, respectively. DCA showed that the nomogram could be applied clinically if the risk threshold was between 29.52 and 99.61% in the training set and between 31.32 and 98.49% in the testing set. RCS showed that when the value of independent risk factors from the predicted model exceeded the median, the mortality hazard ratio increased sharply. The results of the *Hosmer–Lemeshow* test (*χ*^2^ = 0.1901, *df* = 2, *p* = 0.9091) and the calibration curves of the training and validation sets showed good agreement with the actual results, which indicated good stability of the model.

**Conclusion:**

Our nomogram, including CHD, LYMP, NLR, RDW, lactic acid, PT, CRP, PCT, Tbil, ALT, and IL6, exhibits good performance for predicting mortality risk in adult sepsis patients.

## Introduction

Sepsis is a severe inflammatory response associated with high mortality and medical costs worldwide. A pivotal epidemiological survey published in *Lancet* indicated that in 2017, the incidence of sepsis was approximately 48.9 million and caused 11.0 million (10.1–12.0) sepsis-related deaths (1). The latest epidemiological survey in China indicated that sepsis affected one-fifth of patients admitted to ICUs with a 90-day mortality rate of 35.5% (2), and the mortality rate associated with sepsis exhibits an increasing trend globally (3–5).

Early recognition and prompt initiation therapy during sepsis are essential. Based on the severity of sepsis, systemic inflammatory response syndrome (SIRS) progresses to severe sepsis and septic shock, which may be complicated by multiple organ failure, leading to a high mortality rate (6, 7). Establishing a mortality risk prediction for sepsis can help recognize sepsis during its early stage to ensure early clinical intervention and prevent progression into septic shock or multiple organ failure. It is widely acknowledged that with early recognition and prompt therapy, the sepsis mortality rate can decline significantly (8, 9). The past decade has witnessed unprecedented scientific progress. A study by Baysan et al. (10) substantiated that lactate and its clearance play an important role in predicting in-hospital mortality in critically ill patients with sepsis; however, no decision curve analysis was conducted in this research, and the predictive model only underwent internal validation. Zeng et al. (11) developed a novel blending machine learning (ML) model for hospital mortality prediction in ICU patients with sepsis. Nevertheless, model establishment and variable selection were based on logistic regression, and patients with missing data were excluded, suggesting that some potentially valuable variables were not comprehensively analyzed.

Herein, we established a mortality risk predictive model for sepsis based on variables identified by LASSO regression and developed a nomogram with logistic regression. To validate the predictive capability of the nomogram, discrimination and DCA for both the training set and validation set were performed. Finally, the *Hosmer–Lemeshow* test and calibration curve were used for nomogram calibration. Model discrimination is to define a potential cut-off point that distinguish between positive and negative events correctly. Calibration is also as known as consistency or goodness of fit, it reflects the accuracy of the model in predicting absolute risk. DCA is mainly used to determine which intervention measure can maximize the clinical benefits for patients.

## Methods

### Clinical data

The clinical data of this study were obtained by retrieving the electronic medical records of Affiliated Huadu Hospital, Southern Medical University, between 2019 and 2021. A total of 696 patients diagnosed with sepsis according to the Sepsis-3 definition (12) were further screened, all patients were hospitalized due to the primary diagnosis of sepsis. After excluding patients aged <18 years old (*n* = 35), hospitalization time < 24 h (*n* = 17), patients complicated with malignant tumors (*n* = 23), immunosuppression statement (*n* = 18) and clinical data that could not be extracted (*n* = 21), 582 sepsis patients were finally included in our research. Based on the retrospective research of this study, missing values were inevitable. Including 26 variables and 582 clinical cases, the original data theoretically contained a total of 15,132 values. However, 375 missing values arose during the data extraction, and the ratio of missing values was approximately 2.48%. The “*mice*” package from R was used to complete missing data multiple imputation. The “*mice*” package was used in this study because it could design a distribution to obtain reasonable data values according to the specific situation of missing data points.

### Model establishment

**Figure 1:**
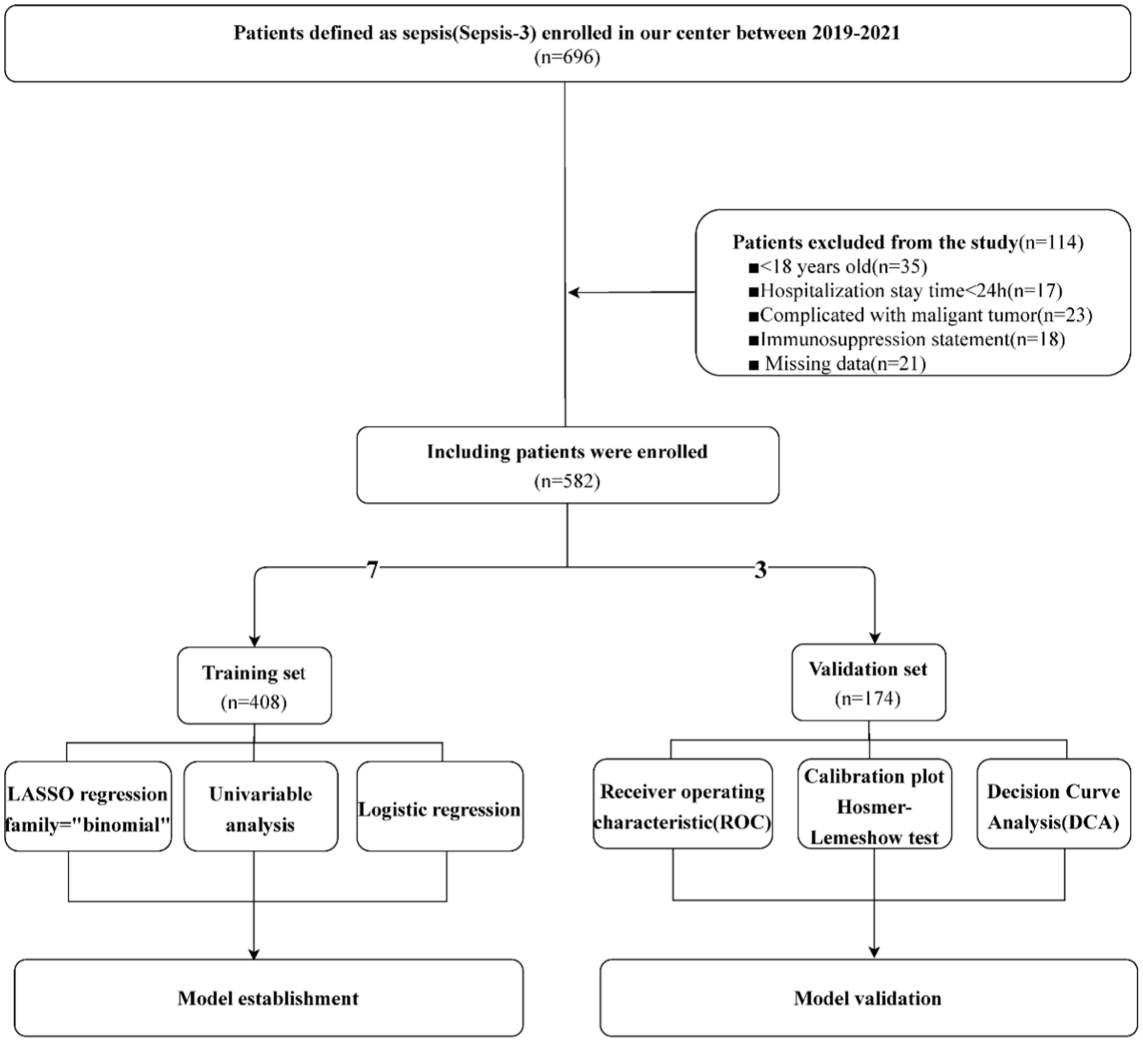
Flowchart of model establishment and validation.

A total of 582 sepsis patients were included in this research and randomly divided into a training set (*n* = 408) and validation set (*n* = 174) at a ratio of 7:3. We extracted the parameters of clinical data such as gender, age, systolic pressure, heart rate and Body Mass Index (BMI), past medical history (diabetes, hypertension, coronary heart disease), evaluation score of severity of sepsis such as Sequential Organ Failure Assessment (SOFA) and Acute Physiology And Chronichealth Evaluation scoring system (APACHE) which obtained within 2 h of sepsis diagnosis, and laboratory tests such as leukocyte count, platelet count, neutrophil count (NEUT), lymphocyte count (LYM), NLR, RDW, procalcitonin (PCT), C-reactive protein (CRP), lactic acid, prothrombin time (PT), international normalized ratio (INR), fibrinogen (FIB), polymers, creatinine (Cr), alanine aminotransferase (ALT), aspartate aminotransferase (AST), total bilirubin (Tbil) and interleukin-6 (IL6) from the electronic medical record system. All laboratory tests mentioned above were performed within 2 h of sepsis onset. To ensure the alarming function and subjective initiative of the models, we abandoned variables generated in late admission and variables regarding treatment. First, LASSO regression was performed for variable selection. The advantage of LASSO regression were as following: (1). Processing high-dimensional data and suitable for feature variable selection problems. (2). Reducing unnecessary feature variables and improve the interpretability and generalization capability of the model. (3). Avoiding the overfitting of the model. Subsequently, binary logistic regression was used to investigate the mortality risk factors from the training set. The independent risk factors identified during binary logistic regression were selected for model establishment and visualized in a nomogram. ROC and DCA curves were generated to verify the nomogram’s prediction ability (13). The core idea of DCA is to compare the clinical benefits of different prediction models under different treatment decision thresholds. It shows the benefits obtained from using predictive models for treatment decision-making under different treatment decision thresholds. Benefits can be defined as the number of cases treated correctly minus the number of cases treated incorrectly, where treatment correctness refers to making correct treatment decisions based on the output results of the predictive model. We also used RCS with five knots at the 5th, 35th, 50th, 65th, and 95th centiles to flexibly model the association of NLR, RDW, lactic acid, PT and IL6 with mortality of sepsis. The merit of RCS is that it makes sure the restriction on boundary conditions, avoiding overfitting of the model and improving model stability during the model construction. Ultimately, we used the *Hosmer–Lemeshow* test and calibration curve for nomogram calibration. The flowchart of clinical data screening, model establishment and model verification was shown in Figure 1.

### Ethics statement

The data collection of this research and implementation were approved by the Ethics Committee of Affiliated Huadu Hospital, Southern Medical University (Registration Number: 2023088). This was a retrospective study. At that time, informed consent was signed because patients needed to be informed that their personal disease data might be included in clinical studies and would not disclose personal privacy. The research was conducted in accordance with the 1964 Declaration of Helsinki and its subsequent amendments.

### Statistical methods

Statistical analysis was performed using R software (4.1.3 Version, R Foundation for Statistical Computing, Vienna, Austria). This was a cross-sectional study whereby the missing values were filled with multiple imputation using “*mice*” package. A total of 582 sepsis patients were randomly divided into a training set (*n* = 408) and validation set (*n* = 174) using the *caret* package at a 7:3 ratio. LASSO regression was performed using the *glmnet* package. The definition of death was mortality during hospitalization. Given that the variable “death” was defined as “present” or “not,” we set its properties as “binomial.” The risk factors were selected as the best predictors by fitted lambda and k-fold (tenfold in this case) cross-validation. In this study, tenfold cross-validation in LASSO regression was used, and only the split ratio of the data set (training set: verification set) was 7:3. In the end, 70% of the data were used for LASSO regression, and 30% were used for verification. Only one verification was carried out. For univariate analysis, Chi-square test used for analysis of differences in discontinuous data. While, for continuous data, if the data conforms to normal distribution, we used *t*-test to compare the differences in mean between the two groups, however if the data did not conform to normal distribution Mann–Whitney U test was used.

From the optimal predictors selected by LASSO regression, the *lrm* function of the *rms* package was used to perform logistic regression. In the present study, we found that a predicted model including history of CHD, LYMP (protective factor), NLR, RDW, lactic acid, PT, CRP, PCT, ALT, Tbil, and IL6 had statistical significance. Finally, a nomogram was generated using the *nomogram* function from the *rms* package.

Furthermore, to investigate the accuracy of the risk prediction model and clinical practicability of the nomogram, ROC and DCA curves were generated using the *pROC* and *ggDCA* packages, respectively. To explore the relationship between the value of each risk factor and its corresponding hazard ratio under nonlinear conditions, we also used the *rms* and *ggplot2* packages for restricted cubic splines (RCS). Finally, with the *calibration* function from the *rms* package, the *Hosmer–Lemeshow* test and calibration curve were conducted for nomogram calibration in the training and validation sets, respectively.

## Results

### Univariate analysis

Among the 582 patients with sepsis including in this study, 182 patients died during the hospitalization in the stage of ICU, the mortality rate of the sepsis patients was about 31.27%. Based on whether the sepsis patient died during hospitalization, univariate analysis was performed. Univariate analysis indicated that gender difference (*χ*^2^ = 12.3, *p* = 0.0005), history of CHD (*χ*^2^ = 22.78, *p* < 0.001), systolic pressure (*t* = −18.898, *p* < 0.001), leukocyte count (*U* = 28,749, *p* = 0.001), platelet count (*U* = 29,389, *p* = 0.0002), neutrophil count (*U* = 26,829, *p* < 0.001), LYMP (*U* = 14,775, *p* < 0.001), NLR (*U* = 15,196, *p* < 0.001), RDW (*t* = 8.219, *p* < 0.001), CRP (*U* = 29,767, *p* = 0.0004), PCT (*U* = 27,399, *p* < 0.001), lactic acid (*U* = 13,335, *p* < 0.001), PT (*t* = 8.612, *p* < 0.001), INR (*U* = 18,959, *p* < 0.001), FIB (*t* = 3.913, *p* < 0.001), D-polymers (*U* = 28,642, *p* < 0.001), Cr (*U* = 29,296, *p* = 0.0002), ALT (*U* = 27,957, *p* < 0.001), AST (*U* = 22,250, *p* < 0.001), Tbil (*U* = 26,197, *p* < 0.001), IL6 (*t* = 19.63, *p* < 0.001), SOFA (*t* = 40.12, *p* < 0.001) and APACHE (*t* = 81.21, *p* < 0.001) between the survival group (*n* = 400) and non-survival group (*n* = 182) were significantly different (Table 1), which showed that all study variables indicated above were univariate risk factors.

**Table 1:** Univariate analysis of sepsis patients with mortality risk.

Factors		Survival (*n* = 400)	Non-Survival (*n* = 182)	*χ^2^/t/U*	*p* value
Gender	Male	202 (50.5%)	121 (66.48%)	12.30	0.0005
	Female	198 (49.5%)	61 (33.52%)		
Age	*x¯* ± *s*	61.85 ± 16.70	64.87 ± 17.58	1.943	0.053
Diabetes	Yes	124 (31%)	60 (32.97%)	0.224	0.6361
	No	276 (69%)	122 (67.03%)		
Hypertension	Yes	157 (39.25%)	85 (46.70%)	2.861	0.0908
	No	243 (60.75%)	97 (53.30%)		
Coronary heart disease	Yes	122 (30.5%)	93 (51.20%)	22.78	<0.0001
	No	278 (69.5%)	89 (48.80%)		
BMI	*x¯* ± *s*	24.29 ± 4.69	24.22 ± 4.70	−0.185	0.853
Systolic pressure (mmHg)	*x¯* ± *s*	130.33 ± 16.76	92.74 ± 24.20	−18.898	<0.0001
Heart rate (time·minute^−1^)	*x¯* ± *s*	118.83 ± 7.86	118.77 ± 7.70	−0.084	0.933
Leukocyte count (×10^9^·L^−1^)	M (P_25_ ~ P_75_)	11.84 (7.34 ~ 16.29)	14.85 (8.10 ~ 23.4)	28,749	0.0001
Platelet count (×10^9^·L^−1^)	M (P_25_ ~ P_75_)	175 (121 ~ 250.5)	145 (62.75 ~ 242.3)	29,389	0.0002
NEUT (×10^9^·L^−1^)	M (P_25_ ~ P_75_)	9.82 (5.75 ~ 14.24)	14.49 (7.51 ~ 21.02)	26,829	<0.0001
LYM (×10^9^·L^−1^)	M (P_25_ ~ P_75_)	1.02 (0.71 ~ 1.52)	0.49 (0.28 ~ 0.73)	14,775	<0.0001
NLR	M (P_25_ ~ P_75_)	9.92 (5.39 ~ 14.69)	26.21 (12.84 ~ 44.95)	15,196	<0.0001
RDW (fl)	*x¯* ± *s*	44.31 ± 7.25	50.48 ± 10.49	8.219	<0.0001
CRP (mg·mL^−1^)	M (P_25_ ~ P_75_)	106.7 (34.73 ~ 162.7)	135.5 (56.51 ~ 193.9)	29,767	0.0004
PCT (ng·mL^−1^)	M (P_25_ ~ P_75_)	9.70 (1.603 ~ 37.0)	19.00 (6.65 ~ 57.86)	27,399	<0.0001
Lactic acid (mmol·L^−1^)	M (P_25_ ~ P_75_)	1.90 (1.25 ~ 3.00)	4.85 (3.25 ~ 9.175)	13,335	<0.0001
PT (S)	*x¯* ± *s*	14.93 ± 2.372	19.03 ± 8.854	8.612	<0.0001
INR	M (P_25_ ~ P_75_)	1.16 (1.063 ~ 1.27)	1.34 (1.18 ~ 1.683)	18,959	<0.0001
FIB	*x¯* ±ss *s*	5.129 ± 1.878	4.449 ± 2.078	3.913	<0.0001
D- polymers (ng/m)	M (P_25_ ~ P_75_)	2,509 (1,244 ~ 4,598)	3,709 (1825 ~ 7,887)	28,642	<0.0001
Cr (umol·L^−1^)	M (P_25_ ~ P_75_)	114.5 (71.85 ~ 232.0)	175.5 (95.00 ~ 313.5)	29,296	0.0002
ALT (U·L^−1^)	M (P_25_ ~ P_75_)	24.50 (15.58 ~ 44.03)	36.55 (19.87 ~ 74.0)	27,957	<0.0001
AST (U·L^−1^)	M (P_25_ ~ P_75_)	26.59 (18.9 ~ 48.00)	57.55 (28.45 ~ 164.3)	22,250	<0.0001
Tbil (μmol·L^−1^)	M (P_25_ ~ P_75_)	13.60 (8.763 ~ 21.42)	19.65 (11.40 ~ 36.50)	26,197	<0.0001
IL-6 (pg·ml^−1^)	*x¯* ± *s*	3.06 ± 1.467	6.06 ± 2.15	19.63	<0.0001
SOFA	*x¯* ± *s*	4.91 ± 2.0	11.66 ± 3.37	40.12	<0.0001
APACHE	*x¯* ± *s*	29.19 ± 7.48	40.79 ± 9.14	81.21	<0.0001

M (P25 ~ P75): M = Median, P25 = Lower quartile, P75 = Upper quartile; *x¯* ± *s*: Mean ± Standard deviation.

### Predictive model establishment

**Figure 2:**
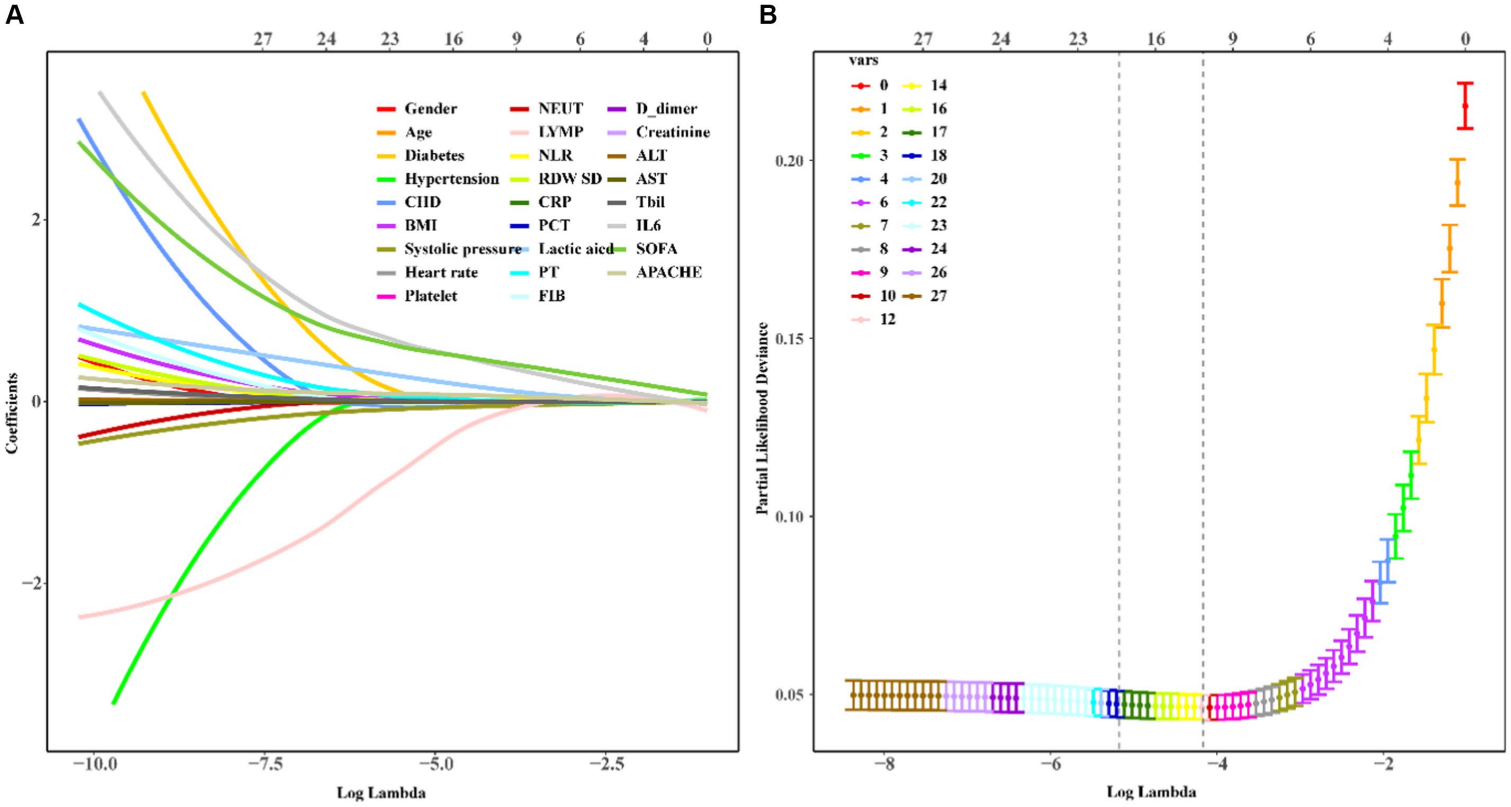
Variable selection by the LASSO regression. A coefficient profile plot was constructed against by the log (lambda) sequence. **(A)** Eleven variables including CHD, NLR, LYMP, RDW, CRP, PCT, lactic acid, PT, ALT, Tbil, and IL6 with nonzero coefficents were selected by deriving the optimal lambda. **(B)** Following verification of the optimal parameter, we used LASSO 1.SE to shrink and select the variables.

**Figure 3:**
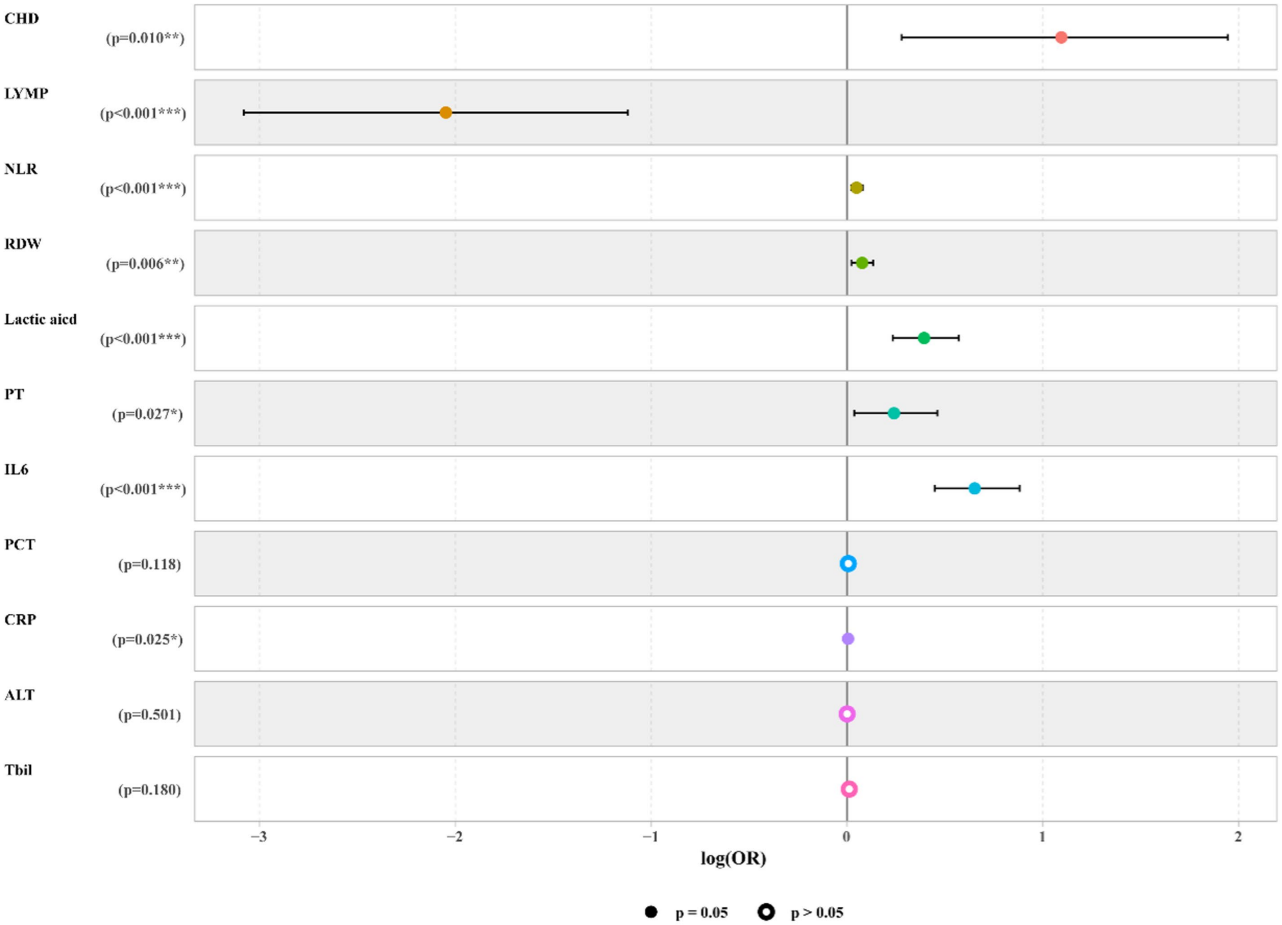
Forest plot of logistic regression model. The forest plot indicated that CHD, NLR, RDW, CRP, PCT, lactic acid, PT, ALT, Tbil, and IL6 were independent risk factors for mortality, while LYMP was a protective factor.

**Figure 4:**
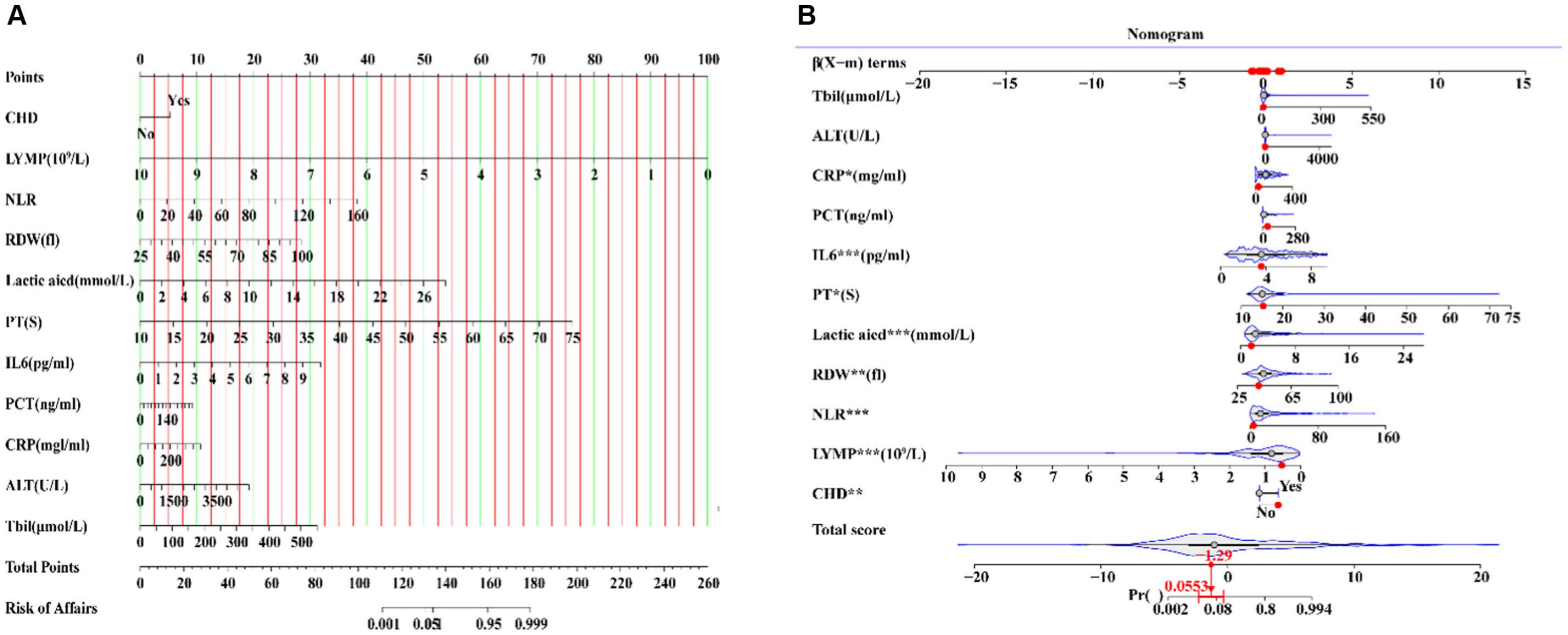
A nomogram including CHD, NLR, LYMP, RDW, CRP, PCT, lactic acid, PT, ALT, Tbil, and IL6 showed mortality risk prediction of sepsis.

We used LASSO regression to select the predictive variables shown in Table 1. Eleven out of twenty-eight variables, including CHD, LYM, NLR, RDW, CRP, PCT, lactic acid, PT, ALT, Tbil, and IL6 were incorporated into binary logistic regression (Figure 2). In order to investigate whether the above including variables involved in collinearity, we used Variance Inflation Factor (VIF) to conduct collinearity analysis. The result showed that all of the VIF from each including variable were less than 10, which implied there was no obvious collinearity among the fitted regression variables (Supplementary Figure S1). Binary logistic regression indicated that CHD, NLR, RDW, CRP, PCT, lactic acid, PT, ALT, Tbil, and IL6 were independent risk factors for mortality, while LYMP was a protective factor. The results of the logistic regression which fitted from the training set are indicated in Figure 3. Moreover, we constructed a nomogram for predicting the mortality risk probability of sepsis patients (Figure 4).

### Predictive model validation and models comparison

**Figure 5:**
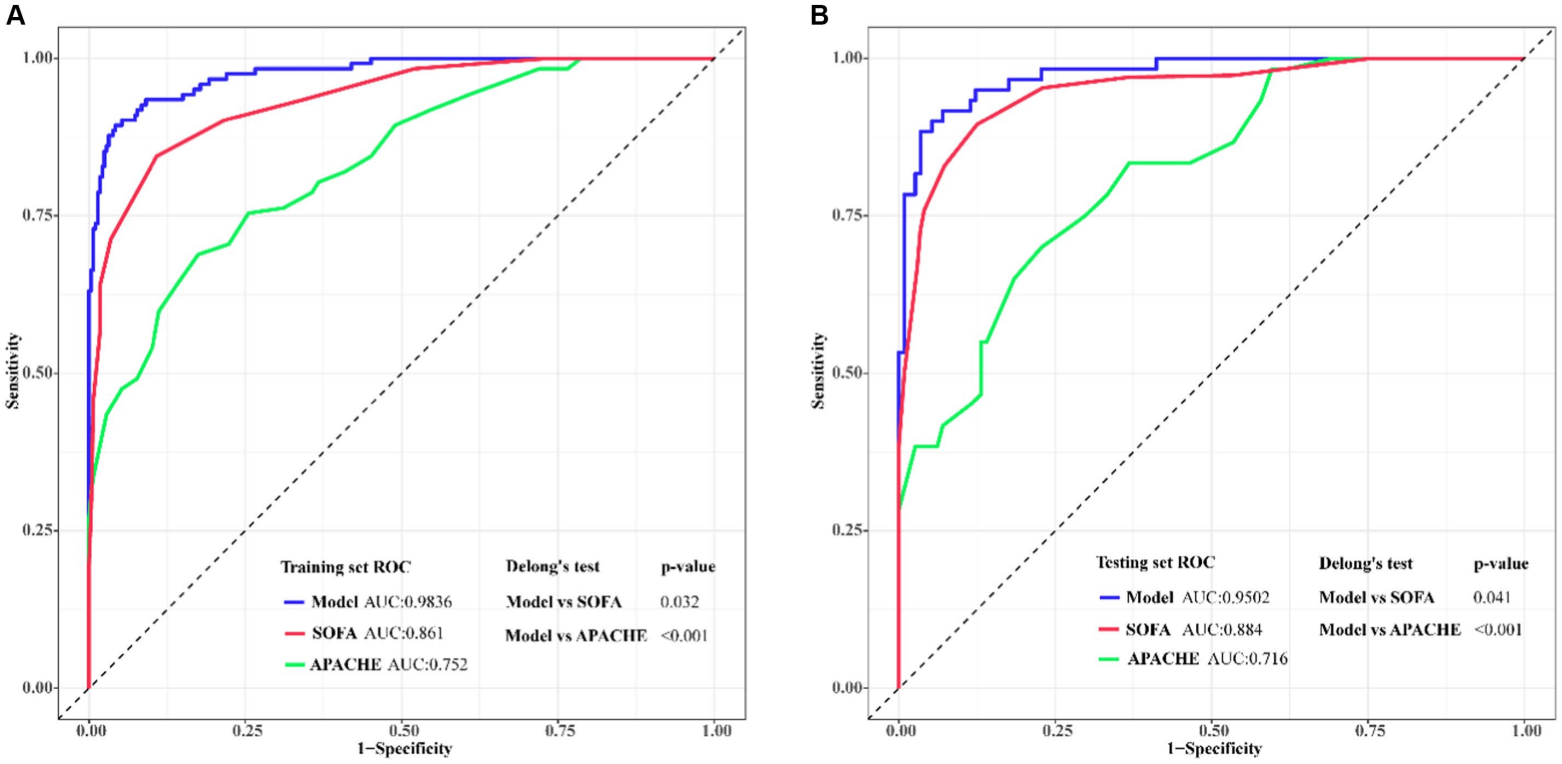
Predictive model validation and models comparison using ROC curves and AUC for adult sepsis. The x-axis represented the specificity of the risk and the y-axis represented the sensitivity of the risk prediction. **(A)** ROCs of training set; **(B)** ROCs of validating set. Compared with traditional SOFA and APACHE scores, the predicted model had larger AUC both in training set and testing set that demonstrated this model had preferable predicted value.

**Figure 6:**
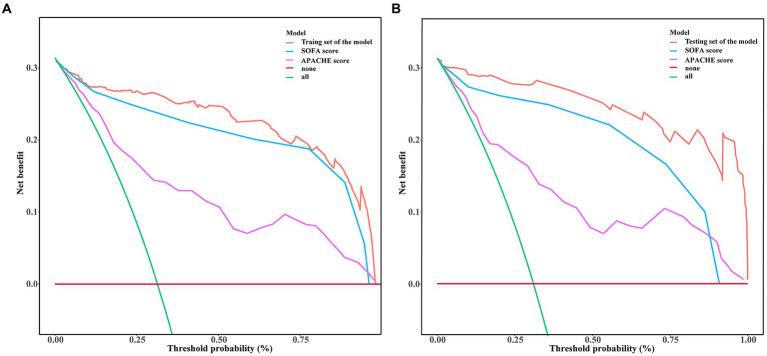
Decision curve analysis (DCA) of mortality risk prediction for adult sepsis among the model and traditional SOFA and APACHE scores. The x-axis represented the risk threshold, and the y-axis measured the net benefit. The DCA curves from either training or testing set showed that the model had higher overall net benefit compared with SOFA and APACHE scores across the majority of the range of reasonable threshold probabilities. **(A)** From training set; **(B)** From testing set.

**Figure 7:**
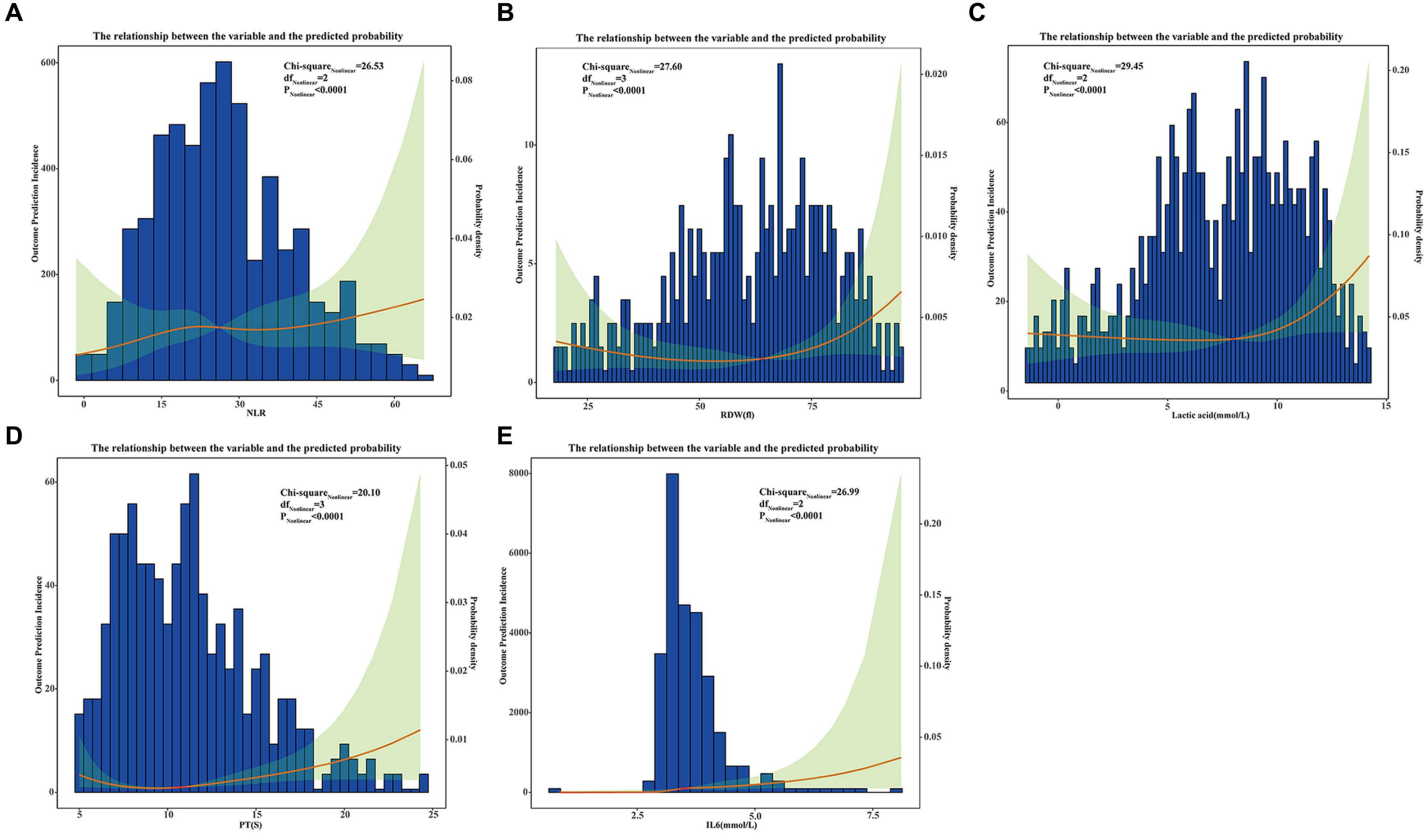
The restricted cubic splines (RCS) to flexibly model and visualize the relation of independent risk factors in nomogram with mortality base on nonlinear correlation. For all of the independent risk factors which may cause mortality including NLR **(A)**, RDW **(B)**, Lactic acid **(C)**, PT **(D)**, and IL6 **(E)**, prediction incidences were indicated by red lines, and their corresponding 95% CIs showed by green shaded areas.

We used ROC curve analysis to validate the diagnostic capability of the predictive model, and we also made a comparison of diagnostic capability among the model and traditional SOFA and APACHE scores. From the training set, the AUC (Area Under Curve) value of the model was 0.9836 (95% CI: 0.9662–1.000), while AUC of the SOFA and APACHE scores were 0.861 (95%CI: 0.7354–0.9221) and 0.752 (95%CI: 0.6877–0.8324) respectively. From the testing set, AUC value of the model was 0.9502 (95% CI: 0.9025–0.9788), while AUC of the SOFA and APACHE scores were 0.884 (95%CI: 0.8127–0.9135) and 0.716 (95%CI: 0.6249–0.8066) respectively. Delong’s test indicated that discrimination between the model and SOFA score had statistical difference in both training set (*p* = 0.032) and testing set (*p* = 0.041), similarly, between the model and APACHE score, the difference were more statistically significant in both training set (*p* < 0.001) and testing set (*p* < 0.001). The ROC curves from our model not only showed the high diagnostic sensitivity and specificity, but also had certain advantages compared to SOFA APACHE scores in diagnostic capability (Figure 5). DCA curves were generated to verify the clinical utility of the model. Our results indicated that the threshold probability of the prediction model in the training set was between 29.52 and 99.61%, while in the testing set, it was between 31.32 and 98.49%, demonstrating valuable clinical efficiency. And the DCA curves from either training or testing set showed that the model had higher overall net benefit compared with SOFA and APACHE scores across the majority of the range of reasonable threshold probabilities (Figure 6). Additionally, considering the nonlinear relationship between dependent variables and independent risk factors, we used restricted cubic splines (RCS) to flexibly model and visualize the relation of independent risk factors in the nomogram with mortality. The results indicated that when the NLR (Median = 23.7214), RDW (Median = 44.7641 fl), lactic acid (Median = 3.6924 mmol/L), PT (Median = 14.5134 S), and IL6 (Median = 6.5924 mmol/L) values exceeded their corresponding medians, the hazard ratio of mortality for sepsis started to increase rapidly (*P* for nonlinearity <0.001) (Figure 7).

### Calibration of the predictive model

**Figure 8:**
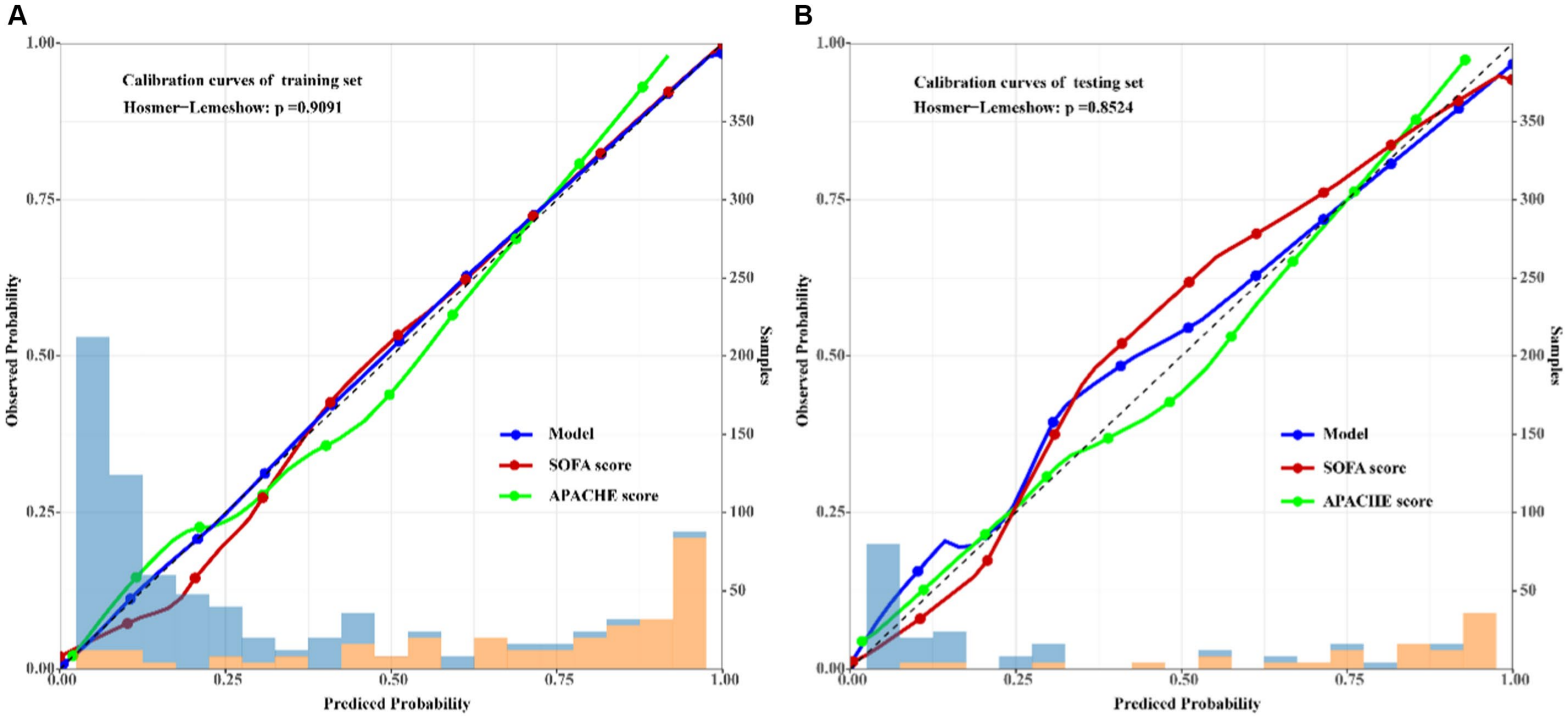
Calibration curves of the predicted mortality risk nomogram of adult sepsis among the model and traditional SOFA and APACHE scores. The x-axis represented the predicted risk of the mortality, the left y-axis represented actual diagnostic cases of the mortality. The diagonal dotted line represented a perfect prediction by ideal model, the solid line represented the performance of the training set **(A)** and testing set **(B)**, the results indicated that compared with SOFA and APACHE, the calibration of model in both training and testing sets were more closer to the ideal reference linear, which indicated good degree of fit of this predicted model.

Furthermore, the results of the *Hosmer–Lemeshow* test (*χ*^2^ = 0.1901, *df* = 2, *p* = 0.9091) indicated that the nomogram of mortality risk prediction exhibited good concordance with the actual results. To visualize the results of the *Hosmer–Lemeshow* test, calibration curves of the model, SOFA and APACHE scores for the training and validation sets were also employed. A closer fit to the diagonal dotted line meant a better prediction (Figure 8).

## Discussion

Given its high mortality rate and complex mechanism, treating sepsis remains a significant conundrum for clinicians (14–16). Early identification and timely therapy are crucial for sepsis to reduce the incidence of sequential organ failure and the mortality rate. Much emphasis has been placed on exploring valuable mortality risk predictive models for sepsis over the past decades (17–20). Nonetheless, the hospital-related costs and high mortality rates caused by sepsis remain significant burdens (21, 22).

According to our mortality risk predictive model for sepsis, eleven indicators, including a history of CHD, NLR, RDW, lactic acid, PT, PCT, CRP, ALT, Tbil, and IL6 were independent predictors of poor prognosis in patients with sepsis, while the LYMP was a protective factor. Nomogram validation using the training and validation sets demonstrated good predictive performance. In addition, internal validation demonstrated the goodness-of-fit and stability of the model. Obviously, compared with traditional SOFA and APACHE score, our predicted model had certain advantage in terms of discrimination, calibration, and decision curve analysis. Although some of the risk factors related to mortality identified in this study were consistent with the literature (23–26), only logistic regression was used for model establishment in these studies. Therefore, good control of confounding factors and variable selection was challenging. Herein, we used LASSO regression to solve this problem and exclude the interference of intermediate variables on the results. Importantly, logistic regression analysis in the present study was based on the linear relationship between the dependent and independent risk variables. We used RCS to visualize the nonlinear relationship between independent risk factors and the hazard ratio of sepsis.

### Association of CHD with mortality risk for Sepsis

Patients often experience insufficient microcirculation perfusion during sepsis or septic shock due to vascular dysfunction. Under such conditions, cardiac output becomes extremely important for maintaining the microcirculation (27–29). An increasing body of evidence (30, 31) suggests that CHD represents a high mortality risk factor for sepsis. Arfaras-Melainis et al. (32) showed that with CHD, the mortality incidence of sepsis was extremely high (may reach 90%). The findings of the present study were consistent with prior studies, which reported that CHD was a mortality risk predictor for sepsis. Accordingly, CHD is a hazard risk factor of mortality for sepsis, protecting and improving cardiac function during the early stages have important clinical significance.

### Association of inflammatory factors with mortality risk for sepsis

Since the third international consensus definition for sepsis and septic shock (Sepsis-3) was held in 2016, an increasing number of studies have pointed out that sepsis should be defined as life-threatening organ dysfunction caused by a dysregulated host response to infection (33, 34). During this pathophysiological process, inflammatory factors such as IL6, TNF-*a*, and neutrophils may trigger an “inflammatory factor storm,” leading to sepsis-related sequential organ failure (26, 35–37). The NLR, which refers to the proportion of neutrophils and lymphocytes, is an indicator of systemic inflammation based on complete blood count values. Once the body experiences severe inflammation, the blood neutrophil count may increase, and lymphocytes decrease accordingly, suggesting that neutrophils represent a risk factor, while lymphocytes are a protective factor (38, 39). In accordance with the literature, our study indicated that the biomarkers IL6 and NLR correlated with a poor prognosis of sepsis.

### Association of RDW with mortality risk for sepsis

RDW represents the variability in the size and form of red blood cells (RBCs) (40). As seen in our model, an increase in RDW indicated a high hazard ratio of mortality. During sepsis or severe sepsis, the potent inflammatory reaction may inhibit the formation and maturation of red blood cells, increase immature red blood cells in the circulation and promote heterogeneity of red blood cells, increasing RDW levels (41, 42). There is a rich literature available substantiating that an abnormal increase in RDW has diagnostic value in sepsis and is predictive of poor prognosis and mortality (43–45). Another advantage of RDW is that it can be easily obtained from routine blood tests and does not need high technical requirements, which is suitable for any primary-level medical center in China.

### Association of lactic acid and coagulation with mortality risk for sepsis

Lactic acid is a metabolite resulting from the fermentation of glucose. When tissues undergo increased anaerobic metabolism, high levels of lactic acid in the blood, sepsis or other diseases can cause death (46, 47). The prothrombin time can be used to reflect human blood coagulation function. Similar to the “lethal triad” (coagulopathy, hypothermia and acidosis) (48), high lactic acid levels and abnormal coagulation indicate a poor prognosis of sepsis. When the level of serum lactic acid increases, the acidic environment may aggravate tissue hypoxia, which can lead to a vicious cycle, and severe tissue hypoxia can prolong prothrombin time and cause coagulation dysfunction. Eventually, DIC or multiple organ failure may occur (49, 50). Overall, dynamic monitoring of lactic acid and PT in the early stage of sepsis is valuable.

### Advance and limitations of this study

This study has a certain degree of advantages. First, the research sampling strategy was rigorous, the risk factors included in the screening were relatively comprehensive. Secondly, in order to make sure that the predictive model does not overfit, LASSO regression was employed variables selection. Thirdly, the including variables of the model are common biomarkers in clinical practice, their capture request not high equipment and technique, and the cost is relatively low. Finally, from internal validation, this model had certain advantages in clinical decision-making compared to traditional SOFA and APACHE scores.

Limitation was inevitable in this study. First, this study was retrospective in nature. Accordingly, potential bias could not be completely excluded. Secondly, the data on sepsis patients were obtained from a single center, and the sample size was relatively small. Thirdly, this study was just based on internal validation and lacked of external validation, we look forward to further applying data from public databases such as the MIMIC database for further validation. In recent years, significant progress has been made in the research of comorbidity index such as Charlson Comorbidity Index or Elixhauser Comorbidity Score, unfortunately, due to the calculation of these comorbidity burden measure involve a series of physiological indicators and psychological assessment questionnaires, which makes it difficult to score in actual clinical practice. Therefore, the data of these comorbidity burden measures could not been collected in this study. And the time span of this study overlaped with the COVID-19 pandemic, due to the institution did not disclose the COVID-19 data, base on that COVID-19 impacted the mortality rates of the sepsis, so there may be more or less bias in the study. Finally, we established a prognostic model based on the independent risk factors in this model. In fact, in actual clinical practice, there are many factors related to the outcome being identified in the model, nevertheless their causal relationship is largely unknown. To solve this problem, Zhang et al. (51) recommended a reliable structural modeling with inverse probability weighting (IPW) to infer causality from observational data, which played an important decision-making role in clinical management. And base on this principle, we expect the causal relationship between risk factors and sepsis are looking forward to investigating in the future.

## Conclusion

Our predictive model, which included six indicators named CHD, NLR, RDW, lactic acid, PT and IL6, yielded good performance for predicting mortality risk in adult sepsis patients.

## Data availability statement

The raw data supporting the conclusions of this article will be made available by the authors, without undue reservation.

## Ethics statement

The studies involving humans were approved by Ethics Committee of Affiliated Huadu Hospital, Southern Medical University. The studies were conducted in accordance with the local legislation and institutional requirements. The participants provided their written informed consent to participate in this study.

## Author contributions

HW: Conceptualization, Formal analysis, Writing – original draft. SJ: Resources, Writing – review & editing. BL: Data curation, Writing – review & editing. TJ: Data curation, Writing – review & editing. JH: Data curation, Writing – review & editing. YL: Data curation, Writing – review & editing. TC: Data curation, Methodology, Writing – review & editing. KM: Funding acquisition, Supervision, Validation, Writing – review & editing.
