# Supplementary material for: Establishment of a mortality risk nomogram for predicting in-hospital mortality of sepsis: cohort study from a Chinese single center
Source: Front Med (Lausanne). 2024 May 3;11:1360197. doi: 10.3389/fmed.2024.1360197 (PMC11100418; doi:10.3389/fmed.2024.1360197)
Supplement: Supplementary file 1 [file Data_Sheet_1.docx]

**Supplementary material of “Establishment of a Mortality Risk Nomogram for Predicting In-hospital Mortality of Sepsis: Cohort Study from a Chinese Single Centre”**


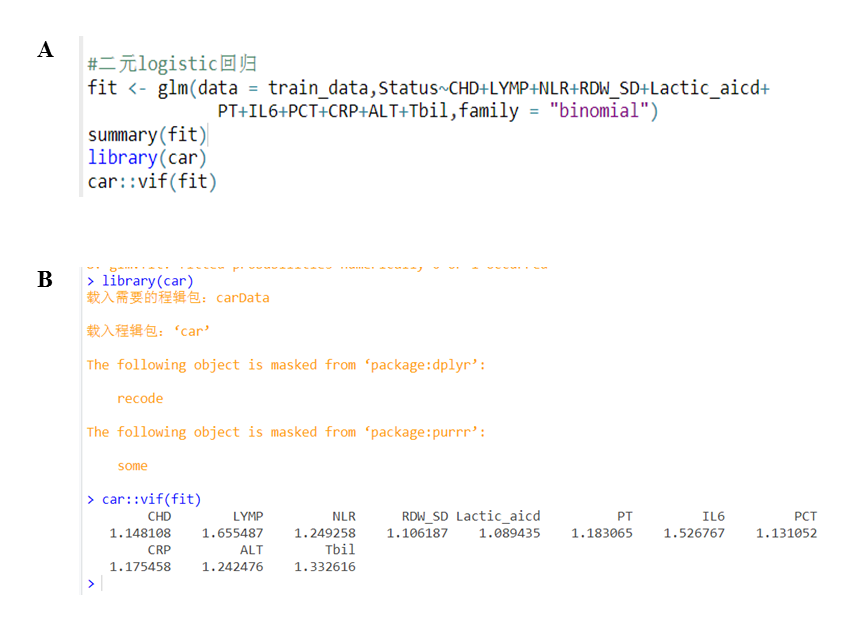


**Supplementary Fig1**: Collinearity analysis of the including variables using Variance Inflation Factor(VIF) method before constructing the logistic regression model. (A). The method and R code for testing the presence of collinearity in the included variables. (B). The result of collinearity analysis. All of the the VIF from each including variable were less than 10, which implied there was no obvious collinearity among the fitted regression variables.


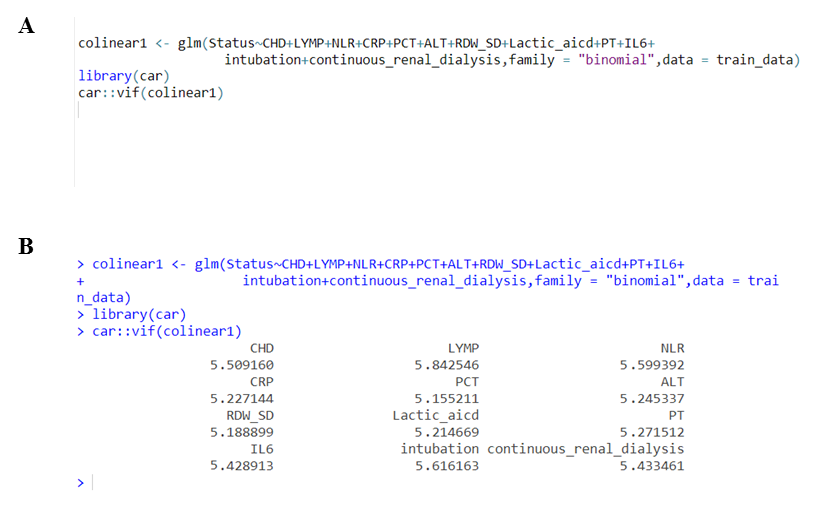


**Supplementary Fig2**: Collinearity analysis of the correlation between the including risk variables and the treatment using Variance Inflation Factor(VIF) method. VIF<5 indicated less collinearity, 5≤VIF<10 indicated moderate collinearity, VIF≥10 indicated obvious collinearity. (A). The method and R code for testing the presence of collinearity in the included variables. (B). The result of collinearity analysis indicated that added the factors such as intubation and continuous renal dialysis, the VIF≥5 implied moderate collinearity.
